# Supplementary material for: Effects of plain water intake before bedtime on sleep and depressive mood among middle-aged Japanese men
Source: PLoS One. 2026 Jan 6;21(1):e0340490. doi: 10.1371/journal.pone.0340490 (PMC12774356; doi:10.1371/journal.pone.0340490)
Supplement: S1 Table — (DOCX) [file pone.0340490.s001.docx]

S1. Results of Tukey's HSD test (*p* values).

| Scores of scales | | Water intake  behavior v.s.,  CES-D score | Thirst in the  morning v.s.,  CES-D score | Water intake  behavior v.s.,  ISI score | Water intake  behavior v.s.,  ISI score | Water intake  behavior v.s.,  Thirst in the  morning |
| --- | --- | --- | --- | --- | --- | --- |
| 1 (Strongly disagree) | 2 | 0.77 | 0.22 | 0.17 | 0.19 | 0.21 |
|  | 3 | 0.57 | < 0.05 | < 0.01 | < 0.05 | < 0.01 |
|  | 4 | 1.00 | < 0.01 | 0.06 | < 0.01 | < 0.05 |
|  | 5 | 1.00 | < 0.01 | < 0.05 | < 0.01 | < 0.05 |
|  | 6 | 0.49 | < 0.01 | 0.92 | < 0.01 | 0.24 |
|  | 7 (Strongly agree) | 0.90 | 0.07 | 0.83 | < 0.01 | 0.74 |
|  |  |  |  |  |  |  |
| 2 | 3 | 1.00 | 0.98 | 0.93 | 1.00 | 0.82 |
|  | 4 | 0.43 | < 0.01 | 1.00 | 0.14 | 1.00 |
|  | 5 | 0.24 | 0.25 | 1.00 | 0.14 | 0.99 |
|  | 6 | < 0.01 | < 0.05 | 0.32 | < 0.05 | 1.00 |
|  | 7 | < 0.05 | 0.59 | 0.90 | < 0.05 | 0.98 |
| 3 | 4 | < 0.05 | < 0.05 | 0.55 | 0.36 | 0.62 |
|  | 5 | < 0.05 | 0.73 | 0.96 | 0.36 | 0.95 |
|  | 6 | < 0.01 | 0.17 | < 0.01 | 0.10 | 0.16 |
|  | 7 | < 0.01 | 0.80 | 0.22 | 0.06 | 0.26 |
|  |  |  |  |  |  |  |
| 4 | 5 | 0.99 | 0.78 | 0.97 | 1.00 | 0.99 |
|  | 6 | < 0.01 | 1.00 | < 0.01 | 0.85 | 0.86 |
|  | 7 | 0.40 | 1.00 | 0.79 | 0.38 | 0.81 |
|  |  |  |  |  |  |  |
| 5 | 6 | < 0.05 | 0.77 | < 0.01 | 0.85 | 0.52 |
|  | 7 | 0.70 | 0.99 | 0.51 | 0.38 | 0.59 |
|  |  |  |  |  |  |  |
| 6 | 7 | 1.00 | 1.00 | 1.00 | 0.92 | 1.00 |

CES-D, the Center for Epidemiologic Studies Depression Scale; ISI, the Insomnia Severity Index.
