# Supplementary material for: Effects of plain water intake before bedtime on sleep and depressive mood among middle-aged Japanese men
Source: PLoS One. 2026 Jan 6;21(1):e0340490. doi: 10.1371/journal.pone.0340490 (PMC12774356; doi:10.1371/journal.pone.0340490)
Supplement: S2 Table — (DOCX) [file pone.0340490.s002.docx]

S2. Results of two-way repeated measures ANOVA for InBody measurements.

|  |  | df | *F* | *p* | *η2* | Hyunh-Feldt |
| --- | --- | --- | --- | --- | --- | --- |
| ECW/TBW |  |  |  |  |  |  |
|  | Time | 1, 54 | 207.1 | 0.00 | 0.79 | 1 |
|  | Condition | 1, 54 | 6.6 | 0.01 | 0.11 | 1 |
|  | Interaction | 1, 54 | 0.1 | 0.76 | 0.002 | 1 |
| ICW |  |  |  |  |  |  |
|  | Time | 1, 54 | 32.7 | 0.00 | 0.38 | 1 |
|  | Condition | 1, 54 | 0.2 | 0.70 | 0.003 | 1 |
|  | Interaction | 1, 54 | 0.03 | 0.87 | 0.001 | 1 |
| ECW |  |  |  |  |  |  |
|  | Time | 1, 54 | 199.8 | 0.00 | 0.79 | 1 |
|  | Condition | 1, 54 | 2.1 | 0.16 | 0.04 | 1 |
|  | Interaction | 1, 54 | 0.2 | 0.66 | 0.004 | 1 |
| TBW |  |  |  |  |  |  |
|  | Time | 1, 54 | 93.2 | 0.00 | 0.63 | 1 |
|  | Condition | 1, 54 | 0.8 | 0.38 | 0.01 | 1 |
|  | Interaction | 1, 54 | 0.01 | 0.94 | > 0.001 | 1 |
