# Supplementary material for: Effects of plain water intake before bedtime on sleep and depressive mood among middle-aged Japanese men
Source: PLoS One. 2026 Jan 6;21(1):e0340490. doi: 10.1371/journal.pone.0340490 (PMC12774356; doi:10.1371/journal.pone.0340490)
Supplement: S3 Table — (DOCX) [file pone.0340490.s003.docx]

S3. Descriptive statistics of InBody measurements.

|  | Control | | Water intake | |
| --- | --- | --- | --- | --- |
|  | Night | Morning | Night | Morning |
| ECW/TBW | 0.4 ± 0.006 | 0.4 ± 0.006 | 0.4 ± 0.007 | 0.4 ± 0.007 |
| ICW | 23.8 ± 2.25 | 23.6 ± 2.28 | 23.8 ± 2.28 | 23.6 ± 2.30 |
| ECW | 14.6 ± 1.35 | 14.3 ± 1.37 | 14.7 ± 1.36 | 14.4 ± 1.40 |
| TBW | 38.4 ± 3.56 | 37.9 ± 3.63 | 38.5 ± 3.60 | 38.0 ± 3.66 |
