# Supplementary material for: Effects of plain water intake before bedtime on sleep and depressive mood among middle-aged Japanese men
Source: PLoS One. 2026 Jan 6;21(1):e0340490. doi: 10.1371/journal.pone.0340490 (PMC12774356; doi:10.1371/journal.pone.0340490)
Supplement: Attachment — Submitted filename: Response to reveiwers.docx [file pone.0340490.s004.docx]

**Author’s responses to the editor and reviewers**

We sincerely thank the academic editor Dr. Yosuke Yamada and anonymous experts for the time spent reviewing our manuscript and providing helpful comments. We have substantially revised our manuscript based on these comments and believe that it is much improved. Our point-by-point responses to each comment are listed below, and changes are shown in red in the revised manuscript. We hope that our manuscript will now be deemed suitable for publication in PLOS ONE.

**Responses to Reviewer 1**

This manuscript investigates the effects of plain water intake before bedtime on sleep parameters and depressive mood in middle-aged Japanese men. The study consists of two parts: a large-scale online survey and a laboratory-based experimental intervention. The research question is highly relevant and of significant public health interest, particularly given the potential for simple behavioral interventions to influence mental health and sleep quality. However, there are several points where the manuscript could be improved to enhance clarity, scientific rigor, and overall readability.

**Comment 1:** There is a contradiction regarding REM sleep latency. The Abstract states that REM sleep latency was prolonged following water intake, whereas the General Discussion states that “plain water intake reduced REM sleep latency.” This is a critical inconsistency and should be corrected throughout the manuscript for clarity and accuracy.

**Response:** We corrected the error in the Discussion section: “Plain water intake prolonged REM sleep latency and reduced the amount of REM sleep.” Page 17, line 12.

**Comment 2:** The manuscript reports numerous t-tests without any mention of correction for multiple comparisons. Given the number of outcomes tested (e.g., sleep parameters, CES-D, PVT), the potential for Type I error is significant. Please discuss whether adjustments (e.g., Bonferroni correction, FDR) were considered.

**Response:** We used only the *t*-test for one-time comparisons of independent data (Figure 5). Multiple comparisons (Figure 1) were assessed using Tukey HSD tests. We believe that Type I error could be avoided by applying the appropriate statistical analyses.

**Comment 3:** Although Cohen’s d values are consistently reported, it would be helpful to interpret the effect sizes in context (e.g., small, medium, large).

While the negative correlation between water intake and CES-D scores in Study 1 is statistically significant (r = -0.18), the effect size is small. The authors should clarify the practical significance of this association and temper conclusions accordingly.

**Response:** Small effect sizes (*η*^2^ = 0.01-0.06) can be explained to some extent by quite large substantial physiological differences such as sweating ability, kidney function, bladder size, and skin surface. Despite the small effect size, we believe that plain water intake is a relatively simple action with the potential to improve depressive mood in the morning. We weakened the conclusion and added a discussion about small effect sizes in the Discussion section. Page 19, line 5-9.

**Comment 4:** Study 2 participants reported CES-D scores averaging around 8, which is lower than the typical mean (~12) in the Japanese population. This suggests the sample was generally mentally healthy, which could limit the generalizability of findings. This limitation should be emphasized more explicitly in the Discussion.

**Response:** We added this as a limitation in the Discussion section: “Our relatively strict selection criteria for participating in the study might have resulted in a cohort of individuals with fairly good mental health status. This might restrict the applicability of our findings to a wider population that is generally mentally healthy.” Page 19, line 12-14.

**Comment 5:** Although the manuscript notes that nocturia frequency increased with pre-bedtime water intake, the clinical risks of nocturia, particularly among older adults (e.g., risk of falls and fractures), should be discussed in greater detail to provide context for the practical implications of this side effect.

**Response:** We added the practical implications of the side effect of nocturia. “Having a bedroom location close to a restroom can help lower accident risks involving nocturia, such as tripping on the floor or falling down the stairs. The paradox of water intake before bedtime requires further exploration.” Page 19, line 1-4.

**Comment 6:** Several sentences could be revised for improved academic English.

**Response:** A professional native English-speaking editor proof-checked our revised manuscript.

**Comment 7**

Some figure legends, such as Figure 5, are rather brief. Adding brief descriptions of the main findings shown in the figures would improve reader comprehension. Some abbreviations (e.g., ECW, TBW) should be defined in figure legends where first used.

**Response:** We added brief descriptions of the main findings to the figure legends and explained all abbreviations that appeared in figure legends. Page 20, Legends.

**Comment 8:** Overall, this is a valuable and well-designed study addressing an underexplored topic of significant public health interest. The combination of observational and experimental approaches strengthens the validity of the findings. The required revisions relate mainly to clarifying inconsistencies, improving statistical reporting, and enhancing the clarity of language and interpretation. With these changes, the manuscript would be a strong candidate for publication in PLOS ONE.

**Response:** We are very much encouraged by this comment.

**Comments from Reviewer 2**

**Comment 9:** If this manuscript is intended to report two studies within a single paper, the current structure—where each study has separate background and discussion sections—makes the paper difficult to follow and disrupts its logical flow. I strongly recommend either:

1.Submitting the studies as two separate manuscripts, or 2.Presenting Study 1 and Study 2 only in the Methods and Results sections, while maintaining unified Introduction, Discussion, and Conclusion sections.

Additionally, the manuscript must conform to the structural format commonly accepted by the journal, namely: Introduction, Materials and Methods, Results, Discussion, and Conclusion. The current structure impairs readability and comprehension.

**Response:** We merged the Introduction and Discussion sections of the two studies.

**Comment 10-1**

Page 14, Line 1 – Table 1

•The reported TST appears short (approximately 300 minutes). Was this due to the unfamiliar hotel environment? Although the Morningness-Eveningness Questionnaire indicated that participants were, on average, of intermediate chronotype, individual variation might have affected sleep duration under experimental conditions.

•Both trials show high WASO; could this also be due to discomfort in the hotel setting or increased nocturnal urination?

**Response:** The reasons for the reduced total amount of sleep in our participants compared to the average reported for Japanese individuals in their 50s (~ 6.34 hours), and high WASO remains unclear. The study was a within-subject design with a counterbalanced condition order, thereby minimizing potential inter-individual variability. Nevertheless, the experiment proceeded during the summer, when seasonal fluctuations in sleep duration (Suzuki *et al.,* 2019, *Plos One*, e0215345) and the unprecedented high temperatures in Japan recorded during the summer of 2024 (Takemura *et al*., 2025, *SOLA*, 329–339) might have been factors. Furthermore, sleeping in a hotel might also have indeed impacted sleep duration. We added this as a limitation in the Discussion section. Page 19, line 16-23.

**Comment 10-2**

•Interestingly, despite the increase in nocturia, WASO did not increase accordingly. This may suggest a novel observation that warrants further discussion.

•The current data also suggest that plain water may lead to rapid excretion through urine, potentially increasing nocturnal urination and mid-sleep awakenings.

•However, WASO did not increase despite increased nocturia—please explain this apparent discrepancy.

**Response:** We believe that the time taken to use the bathroom did not significantly affect WASO. The discrepancy between the increase in nocturia but not in WASO can be explained by the fact that the difference in frequent nocturia was not so large between the experimental and control conditions (0.70 ± 0.439 *vs*. 0.24 ± 0.262, respectively). The small amount (280 mL) of water intake before sleep might not be sufficient to affect WASO, even though it slightly increased the frequency of nocturia. We added this point to the Discussion section. Page 18, line 17-21.

**Comment 10-3**

•Was wake-up time standardized? Did participants truly secure 7.5 hours of sleep as suggested in the figure?

**Response:** Figure 3 shows the standard schedule planned in the experiment protocol, and it does not mean that the participants had 7.5 hours of sleep. The total sleep time was around 300 min and differed among participants within the ranges shown in Table 1. The time was measured by polysomnography.

**Comment 11:** Page 11, Line 4 – “average water loss during sleep (400–700 mL)”

•It is unclear how this estimate justifies the choice of 280 mL of plain water before bedtime. Please clarify the rationale behind selecting this amount.

**Response:** The reported average water loss during sleep is 400–700 mL, which could cause nocturia. Considering nocturia, we set the water intake amount to 280 mL based on the average amount of water lost during 2 hours before bedtime (Yamada *et al*., 2022). We explained the rationale in the Methods section. Page 10, line 4-6.

**Comment 12:** Page 17, Line 19 – General Discussion

•The discussion focuses on concerns about dehydration, but it is unclear—both from the methods and the background—why plain water was selected as the rehydration fluid.

•If dehydration mitigation was a concern, previous studies have shown that beverages containing electrolytes and/or carbohydrates are more effective for hydration maintenance or recovery.

**Response:** Indeed beverages containing electrolytes and/or carbohydrates are more effective for maintaining or recovering hydration than plain water. We aimed to obtain fundamental information about the impact of rehydration immediately before sleep on depressive mood in older adult men after waking up in the morning. This is first investigation of this topic as far as we can ascertain. We therefore tested our hypotheses using plain water because it is the most basic beverage consumed by humans for rehydration compared with beverages containing electrolytes and/or carbohydrates. This matter is described in the Discussion section. Page 17, line 8-11.

**Comment 13-1**

Page 19, Line 2 – REM Latency and REM Sleep

•REM latency increased and REM duration decreased. Would such changes positively influence the clearance of neural waste products or psychological recovery during sleep?

**Response:** A study of mice (Tsai et al., 2021) found that REM sleep might be associated with the clearance of neural waste substances, but this has not been proven in humans. Therefore, we mentioned this as a possible cause of reduced depressive mood in the participants who consumed water compared to those who did not.

**Comment 13-2**

•Line 57 seems to suggest the opposite. If I have misunderstood, I apologize—but clarification is needed as this appears contradictory.

Page 17, Line 26 – Statement: “We determined that plain water intake reduced the amount of REM sleep and increased REM sleep latency.”

•This conclusion seems inconsistent with the overall claims of the study. Please ensure alignment between the data and key conclusions.

**Response:** We corrected this error.

**Comment 14:** Figure 4 – ECW/TBW Differences

•The difference in ECW/TBW ratios raises concern about experimental control. Although the study appears to have been carefully conducted, could the timing or content of pre-sleep meals or trial food have influenced these values?

**Response:** During the experiment, three meals and anything else consumed before sleeping were controlled under both conditions, so we believe that they did not affect the difference in ECW/TBW. We assume that the difference in ECW/TBW before sleep between the conditions was due to habituation from consecutive nights of water intake over a week. We added this as a limitation to the Discussion section.

**Comment 15-2**

•Please include discussion of potential confounding factors, including any interaction effects.

**Response:** Physiological inter-individual differences such as sweating ability, kidney function, bladder size, and surface skin area could be latent confounding factors. This is mentioned in the Discussion section.

**Journal Requirements**

1. Please ensure that your manuscript meets PLOS ONE's style requirements, including those for file naming.

**Response:** We changed the file names to meet the style requirements of PLOS ONE.

2. We note that the grant information you provided in the ‘Funding Information’ and ‘Financial Disclosure’ sections do not match. When you resubmit, please ensure that you provide the correct grant numbers for the awards you received for your study in the ‘Funding Information’ section.

**Response:** We corrected the mismatch between “Funding Information” and “Financial Disclosure” removed funding-related text from the manuscript, and included the amended statements in our cover letter.

3. Thank you for stating the following in the Acknowledgments Section of your manuscript:

We are grateful to Kiyoshi Yoshinaka, Naotaka Nitta, Mototada Shichiri, Hiroshi Endo, Sayaka Higo-Yamamoto, Chisa Emura for critical discussions. We thank CPCC Co., Ltd. (Tokyo, Japan) for conducting the clinical trial. We thank Suntory Beverage & Food Limited for financial support and Hiroyuki Kato, Susumu Takemoto, Yasuhiro Tanaka for helpful comments regarding the experimental protocol and this draft of the manuscript.

We note that you have provided funding information that is not currently declared in your Funding Statement. However, funding information should not appear in the Acknowledgments section or other areas of your manuscript. We will only publish funding information present in the Funding Statement section of the online submission form.

Please remove any funding-related text from the manuscript and let us know how you would like to update your Funding Statement. Currently, your Funding Statement reads as follows:

The study was funded by Suntory Beverage & Food Limited (Tokyo, Japan).

Please include your amended statements within your cover letter; we will change the online submission form on your behalf.

**Response:** We removed funding-related text from the manuscript and included the amended statements within our cover letter.

4. We note that you have indicated that there are restrictions to data sharing for this study. For studies involving human research participant data or other sensitive data, we encourage authors to share de-identified or anonymized data. However, when data cannot be publicly shared for ethical reasons, we allow authors to make their data sets available upon request. For information on unacceptable data access restrictions, please see http://journals.plos.org/plosone/s/data-availability#loc-unacceptable-data-access-restrictions.

Before we proceed with your manuscript, please address the following prompts:

a) If there are ethical or legal restrictions on sharing a de-identified data set, please explain them in detail (e.g., data contain potentially identifying or sensitive patient information, data are owned by a third-party organization, etc.) and who has imposed them (e.g., a Research Ethics Committee or Institutional Review Board, etc.). Please also provide contact information for a data access committee, ethics committee, or other institutional body to which data requests may be sent.

b) If there are no restrictions, please upload the minimal anonymized data set necessary to replicate your study findings to a stable, public repository and provide us with the relevant URLs, DOIs, or accession numbers. Please see http://www.bmj.com/content/340/bmj.c181.long for guidelines on how to de-identify and prepare clinical data for publication. For a list of recommended repositories, please see https://journals.plos.org/plosone/s/recommended-repositories. You also have the option of uploading the data as Supporting Information files, but we would recommend depositing data directly to a data repository if possible.

Please update your Data Availability statement in the submission form accordingly.

**Response:** We uploaded the data as Supporting information files.

5. Please ensure that you refer to Figure 4 in your text as, if accepted, production will need this reference to link the reader to the figure.

**Response:** We refer to Figure 4 in the text. Page 13, line 10.

6. Please include captions for your Supporting Information files at the end of your manuscript, and update any in-text citations to match accordingly. Please see our Supporting Information guidelines for more information: http://journals.plos.org/plosone/s/supporting-information.

**Response:** We included captions for the Supporting Information files at the end of our manuscript. Page 21, line 14-15.

7. If the reviewer comments include a recommendation to cite specific previously published works, please review and evaluate these publications to determine whether they are relevant and should be cited. There is no requirement to cite these works unless the editor has indicated otherwise.

**Response:** N/A
