# Supplementary material for: Effects of plain water intake before bedtime on sleep and depressive mood among middle-aged Japanese men
Source: PLoS One. 2026 Jan 6;21(1):e0340490. doi: 10.1371/journal.pone.0340490 (PMC12774356; doi:10.1371/journal.pone.0340490)
Supplement: Attachment — Submitted filename: Response_to_reveiwers_auresp_2.docx [file pone.0340490.s005.docx]

**Author’s responses to the editor and reviewers**

We sincerely reiterate our gratitude to the Academic Editor, Dr. Yosuke Yamada, and the anonymous reviewers for the time and effort devoted to the evaluation of our manuscript and for their constructive and insightful comments. Our detailed, point-by-point responses to each remark are presented below, and all revisions have been highlighted in red in the revised manuscript. We believe that, in its current form, the manuscript will now be considered suitable for publication in PLOS ONE.

**Responses to Reviewer 1**

The authors have addressed all prior reviewer and editorial concerns thoroughly.

Major issues such as (1) inconsistency in REM sleep latency, (2) statistical transparency, (3) data availability, and (4) funding statement alignment have been satisfactorily resolved. The revised version demonstrates careful editing and a clear methodological rationale.

The manuscript now meets the criteria for publication in PLOS ONE. The scientific quality is sound, and the conclusions are appropriately cautious and supported by the data. Minor textual polishing may still be needed (e.g., standardizing expressions of “trend” to “marginally significant”), but these do not affect the overall integrity of the work.

This revised version reflects substantial improvement. The writing is clear, the structure coherent, and the key revisions have resolved the previous methodological and interpretive concerns. I appreciate the authors’ careful attention to detail and transparent responses to every comment.

* The contradiction regarding REM sleep latency and duration has been corrected and now aligns with the reported findings.

* The Discussion appropriately acknowledges confounding factors (hydration status, ECW/TBW differences, seasonal and environmental effects).

* Statistical methods and figure legends have been clarified; multiple comparison procedures are now explicitly described.

* Funding, ethics, and data availability statements fully comply with PLOS ONE requirements.

**Comment 1:**

Statistical phrasing: Replace “trend” with “marginally significant (p = …)” for clarity and to conform to journal style.

**Response:**

We employed the term “marginally significant” to describe the statistical outcome. See page 11, line 26.

**Comment 2:**

Conclusion tone: The last paragraph (“advantages and disadvantages”) could end with a more forward-looking statement, such as: Further studies including participants with depressive symptoms are warranted to generalize the findings.

**Response:**

We appended the statement, “Further studies including participants with depressive symptoms are warranted to generalize the findings.” to the concluding sentence. Thank you for your insightful suggestion. See page 20, line 11-12.

**Comment 3:**

Language consistency: Consider minor stylistic edits (e.g., ameliorated depressive mood → “improved morning mood”) for smoother readability.

**Response:**

We revised the phrase “ameliorated depressive mood in the morning” to “improved morning mood” to enhance readability. See page 15, line 8-9.

**Comment 4:**

Data statement: Confirm that Supporting Information files correspond exactly to the datasets referenced in the Results.

**Response:**

We reviewed the Supplement Tables and revised the table nomenclature presented in the Results section. See page 13, line 11.

**Comment 5:**

Overall, this version is scientifically sound and clearly presented. I commend the authors for their comprehensive and thoughtful revision.

**Response:**

Thank you very much for your constructive comments, which have strengthened our manuscript.

**Responses to Reviewer 2**

**Comment 1:**

I have no further major comments. However, I noticed that the number of subjects in the manuscript is slightly different from the UMIN registration (UMIN000054134, Registration Date: 05/01/2024). May be 62.

**Response:**

The initial planning phase involved 60 participants. Owing to attrition during the trial, the cohort was expanded to 63 individuals. At the data analysis stage, missing data reduced the final sample size to 55 participants. Thank you for your comment.
